# Improved process intermediate stability through the identification and elimination of reactive glycation residues – a monoclonal antibody case study

**DOI:** 10.1080/21655979.2022.2086350

**Published:** 2022-06-27

**Authors:** Allen Bosley, Kimberly Cook, Shihua Lin, David Robbins

**Affiliations:** a Purification Process Sciences, AstraZeneca, Gaithersburg, Maryland, USA; b Antibody Discovery & Protein Engineering, AstraZeneca, Gaithersburg, Maryland, USA; c Analytical Biotechnology, AstraZeneca, Gaithersburg, Maryland, USA

**Keywords:** Glycation, stability, charge variants, process intermediate

## Abstract

The manufacturing of therapeutic biologics can result in a heterogeneous population of charge variants, encompassing many quality attributes which could impact activity and pharmacokinetics. Monitoring the relative abundance of these charge variants to demonstrate process consistency is an expectation of regulatory agencies. Control of the relative abundance of charge variants is also necessary to ensure product comparability across the product lifecycle. We have observed a significant shift in the relative abundance of charged species, as measured by capillary isoelectric focusing, during clarified cell culture fluid holds for several monoclonal antibodies. This lack of stability requires that the hold time for this process intermediate be significantly curtailed, eliminating manufacturing flexibility. We have identified the cause of this shift in relative abundance of charged species as changes in glycation levels, focused predominantly on three conserved, solvent accessible, lysine residues. Mutants of a model protein were generated that show increased charge state stability can be gained by eliminating these reactive lysines. Further, characterization studies were conducted on these mutants to determine the impact to biological activity and stability of the molecule, with no detrimental effects observed. Incorporating this knowledge into the assessments of candidate drugs could allow for the selection of molecules less susceptible to this product degradation pathway, allowing for greater manufacturing flexibility. This process of identifying and removing reactive lysine residues could be useful in the design of drug candidates with improved charge state stability, across a range of modalities.

## Graphical abstract

**Figure:**
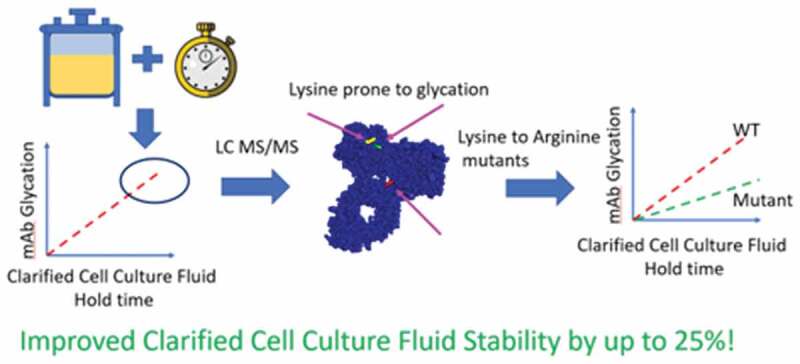

## Highlights

- Observed a shift in the charged species when holding clarified cell culture fluid
- The cause of this shift has been identified as changes in glycation
- Three conserved, solvent accessible, lysines were identified as glycation sites
- Stability was improved by replacing these lysine residues with arginine
- Eliminating glycation sites in drug candidates can result in more stable molecules

## Introduction

During the manufacturing of therapeutic monoclonal antibodies (mAbs) these proteins are susceptible to a host of enzymatic and non-enzymatic modifications. Potential modifications include glycosylation, N-terminal glutamine cyclization, aspartate isomerization, C-terminal lysine processing, deamidation, oxidation, glycation, peptide bond cleavage, and disulfide bond reduction and formation [1]. These modifications occur in the production bioreactor during protein expression [2] but modifications can also occur under conditions experienced during antibody purification [3], and storage under final formulation conditions [4,5]. Regulatory agencies recognize the heterogeneous nature of biological therapeutics and have issued guidance on characterizing and monitoring post-translational modifications. According to The International Council for Harmonization of Technical Requirements for Pharmaceuticals for Human Use (ICH) Q6B ‘The manufacturer should define the pattern of heterogeneity of the desired product and demonstrate consistency with that of the lots used in preclinical and clinical studies.’[6] One method to demonstrate product and process consistency is through monitoring the charge variant distribution through capillary isoelectric focusing (cIEF) as many of the modifications described above impact the isoelectric point of the protein variant.

Glycation is a post-translational modification that has the potential to significantly alter the charge variant distribution, resulting in lot-to-lot inconsistencies, in part because it is difficult to control. Glycation is the non-enzymatic addition of a sugar molecule to a free amino group on a protein. The reaction can occur both in-vivo [7] and in-vitro [8] and the extent of glycation can be impacted by cell culture conditions, such as glucose levels [8] and the mean residence time of excreted protein in the cell culture media.

Previous studies have used enrichment techniques to separate the mAbs glycated during the cell culture process and used this material to identify glycation sites. Quan et al. [9] utilized this method of enrichment to identify eight reactive lysines that appeared to have a different susceptibility to glycation. Here, we were able to identify glycation sites and relative abundance without the need to enrich the sample. This allows for an unbiased comparison between samples and allows for the identification of those sites that are glycated over the course of clarified cell culture fluid hold times.

In another study, Miller et al. [10] looked at a mAb that was highly reactive to glycation, with upwards of 80% of the molecules containing at least one glycation site upon incubation in the presence of glucose. They determined that the bulk of the glycation was occurring at a single site in the Complementarity-determining region. While this modification did not impact potency for this particular molecule, the large lot-to-lot variably in charge variant distribution raised significant process consistency concerns.

While glycation can form in the bioreactor, it can also form after harvest as long as sufficient sugar is present. Glycation can also occur outside the bioreactor so long as there is sufficient reducing sugar present. One example would be drug product formulations that contain sucrose which could hydrolyze into its reducing constituents (glucose and fructose). For this reason, Fischer et al. explored the composition of the drug product formulation on mAb glycation levels[11]. They found products stored in sucrose containing formulations to be at no greater risk for protein glycation.

Another source of variability in overall glycation levels frequently encountered in the manufacturing of monoclonal antibodies is the amount of time between harvest and execution of the capture chromatography step and the conditions under which this material is stored. This time between harvest and capture is defined as the clarified cell culture fluid clarified cell culture fluid (CCCF) hold time and is one of many process intermediate hold times defined as part of the manufacturing process. Process intermediate hold times can be a significant source for product degradation and must be well controlled to ensure product consistency.

We hypothesized that the primary source of changes in charge variant distribution is glycation of conserved lysine residues and that improved stability can be achieved by identifying and removing these reactive lysines. In this work, we studied the impact of the clarified cell culture fluid hold time and temperature on charge variant distribution for several therapeutic monoclonal antibodies. The glycation levels, as determined by Time-of-flight Mass Spectrometry, at various time points were established and compared to the charge variant distributions as determined by Capillary Isoelectric Focusing. Material from the initial and final initial time points of each study were analyzed through peptide mapping to identify those amino acids that were glycated under CCCF hold conditions. Sequence alignment between the test molecules identified several conserved lysine residues that were susceptible to glycation during CCCF storage. Mutants were generated that replaced these lysine residues with arginine to determine if stability could be improved, thereby improving process consistency and accommodating less restrictions on in-process hold times. Additionally, further characterization studies were conducted on the most stable mutant to 1) determine if overall glycation was indeed reduced, 2) assess impact on potency and 3) determine if any other stability indicating attributes are affected. Through this work, we show that hold times can contribute significantly to lot-to-lot variability, in some instances up to 1.5% change in cIEF analysis over the course of a single 24-hour period, and that target mutations to the conserved region can minimize this impact by eliminating sites prone to glycation. Elevated glycation levels were observed on complementarity-determining region lysine residues and we propose that similar methods described here should be used to identify molecules prone to glycation while screening potential drug candidates to prevent stability concerns from arising in later development stages of the molecule.

## Materials and methods

### Clarified cell culture fluid stability studies

CCCF was manufactured in bioreactors using standard technology for monoclonal antibody production. mAb1 was acquired from a 12,000 L manufacturing facility and mAb2 was acquired from a pilot scale (50 L) facility. CCCF containing mAb3 and mAb 3 mutants were generated using lab scale (2 L) bioreactors. CCCF was 0.2 μm sterile filtered and aseptically transferred into sterile and clean 500 mL stainless steel containers. Before use, stainless steel containers were sanitized and cleaned by 1 N NaOH by soaking for at least one hour. Following sanitization, drained stainless-steel containers were rinsed at least three times with USP water prior to steam sterilization for at least 30 minutes and a 20 minute drying cycle in autoclave safe pouches. The stainless-steel vessels containing the CCCF were stored at either 4°C, 12°C, 22°C or 37°C and 50 mL samples were aseptically collected at defined time points. These samples were stored at −80°C prior to being subjected to Protein A chromatography capture.

*Protein A Capture –* Many of the analytical techniques used to assess product quality require that the mAb be purified through a capture step. To accommodate this analysis, CCCF stability study time point samples underwent Protein A capture using MabSelect SuRe (GE Healthcare – Life Sciences, P/N: 17,543,802). The bound material was re-equilibrated, washed, and eluted at pH ~3.5. The resulting product was neutralized to pH ~7.0 prior to storage at −80°C.

### Capillary isoelectric focusing

Capillary electrophoresis was performed as described previously in Cao et al. [12] and Kim et al. [13]. Briefly, *all* mAb1 samples were normalized to 0.25 mg/mL. The pI markers used for this analysis were 5.85 and 9.77. The focus time was set to 5 minutes. Samples were analyzed using the iCE280 (ProteinSimple, #101282) with the FC-Coated Cartridge (ProteinSimple, #101701). Raw data was exported into Empower (Waters Corporation) for analysis.

All mAb2 and mAb3 samples were normalized to 10 mg/mL for mAb2 and 2 mg/mL for mAb3 and digested with Lyophilized Carboxypeptidase B (Millipore, #217356) for 10 minutes at 37°C. Samples were then spun down and normalized to a final concentration of 0.25 mg/mL for mAb1 and 0.5 mg/mL for mAb 2. Two pI markers were added to each sample and samples were cooled to 4°C while sitting on the autosampler. cIEF focus time was set to 5 minutes for mAb1 and 10 minutes for mAb2. Samples were analyzed using the iCE280 (ProteinSimple, #101282) with the FC-Coated Cartridge (ProteinSimple, #101701). Raw data was exported into Empower (Waters Corporation) for analysis.

### Peptide mapping with mass spectrometry

Peptide mapping with mass spectrometry was performed as described previously in Cao et al. [12] and Kim et al. [13]. Briefly, the amino acid sequence and post-translational modifications are confirmed by peptide mapping using a Hybrid Ion Trap-Orbitrap or Orbitrap Fusion mass spectrometer in conjunction with a UPLC/UV system. Denatured samples are reduced and alkylated prior to buffer exchange. The buffer-exchanged samples are digested with trypsin for 4 hours followed by acidic quench. The digested samples are separated using a C18 column (1.7 μm, 300 Å, column dimensions of 2.1 × 150 mm). The identity of the tryptic peptides is confirmed by comparison to the theoretical masses of corresponding peptides (including amino acid composition and post-translational modifications) using MS and MS/MS data. UV profile results were compared to reference standards to assess consistency.

### Time-of-flight mass spectrometry (intact mass analysis)

Time-of-flight mass spectrometry was performed as described previously in Harris et al. [14] and Kim et al. [13]. Briefly, Quadrupole time-of-flight (Q-TOF) mass spectrometry is used to confirm the primary structure of mAb3 based on the accurate measurement of its molecular mass. The samples are prepared with and without DTT reduction. The measured mass of intact or reduced molecule is obtained through the spectral mass deconvolution using a maximum entropy deconvolution software package. Deconvoluted mass is compared to the theoretical mass based on the amino acid composition. Results are reported in Dalton units of molecular mass (Da).

### Generation of monoclonal antibody mutants

All Glycation mutants were assembled individually using PCR by overlap extension with High Fidelity PCR Supermix (Invitrogen) and cloned into a mammalian expression vector with unique restriction sites. DNA for transfection was prepared using the Qiagen Plasmid Plus Maxi Prep Kit. Chinese hamster ovary (CHO) cells were transiently transfected at a density of 2.1 × 10^6^/mL with DNA constructs using PEI (Polyethyleneimine) diluted in 150 mM NaCl and following standard transfection protocol. Transfected CHO cells were fed every 48 hours with enriched media. Cells were harvested and supernatant was collected 14 days post transfection for analysis.

### High performance size-exclusion chromatography

High Performance Size-Exclusion Chromatography (HPSEC) was performed as described previously in Harris et al. [14] and Kim et al. [13]. Briefly, High-Performance Size-Exclusion Chromatography separates molecules on the basis of molecular size and shape using a column containing a rigid, porous silica gel. Larger molecules pass through the column as they are not able to access all of the volume within the stationary phase pores, therefore eluting first. Smaller molecules take a longer path through the gel pores and therefore take longer to elute. The resulting elution profile is monitored for optical absorbance at 280 nm and the data expressed as a relative percentage of monomer, aggregate and low molecular weight fragments.

### Capillary electrophoresis using Agilent bioanalyzer

Capillary electrophoresis was performed as described previously in Kim et al. [13]. Briefly, fragment analysis using the Protein 230 Bioanalyzer kit Agilent Technologies, #5067-1517 was conducted as described in the manufacture guidance document. Briefly, all samples were normalized to 4 mg/mL using 1X PBS (Gibco, #14190-136). Then, 5 mL of the 4 mg/mL sample was added to 5 mL of either non-reducing sample buffer or reducing sample buffer (containing DTT) and heated at 80°C for several minutes. Following spin-down, 6 mL of this 2 mg/mL sample was then added to 84 mL of water. Of this final solution, 6 mL was placed into a well on the Protein 230 microfluidic chip. A total of 10 µL of the provided Protein Ladder matrix (Part II, Agilent Technologies, #5067-1517) was utilized. The LabChip was analyzed using a 2100 Bioanalyzer Instrument (Agilent Technologies, # G2939BA).

### *Microneutralization activity* assay

The activity of mAb3 on neutralizing the cytotoxicity induced by Respiratory Syncytial Virus is measured using a microneutralization assay. This assay measures the mAb3s ability to protect HEp-2 cells from Respiratory Syncytial Virus infection-induced cell death, which can be measured by quantifying ATP level in HEp-2 cells using Cell Titer-Glo^TM^ Assay System (Promega). Ten serial 1.8-fold dilutions of mAb3 were pre-incubated with Respiratory Syncytial Virus at room temperature for 20–60 minutes in a 96-well plate. Then, Hep-2 cells at 20,000 cells/well were added to the plate followed by 48–50 hours incubation at 37°C. At the end of incubation, 100 µL/well of Cell Titer-Glo was added, and the plates were shaken for 60 minutes at room temperature. The amount of luminescence that is proportional to the amount of live cells was measured using an Envision plate reader. The dose response curve was fitted with a 4-parameter model using SoftMax Pro software. The bioactivity of the mAb3 test sample was determined by dividing the IC50 value of reference standard by the IC50 value of test sample and multiplying by 100.

## Results and discussion

We studied the impact of the clarified cell culture fluid hold time and temperature on charge variant distribution for several therapeutic monoclonal antibodies. We identified several conserved lysine residues that were susceptible to glycation during CCCF storage. Mutants were generated that replaced these lysine residues with arginine, which demonstrated improved stability, thereby improving process consistency and accommodating less restrictions on in-process hold times. We also conducted characterization studies on the most stable mutant and determined overall glycation was indeed reduced, this mutation had no impact on potency and no other stability indicating attributes are affected.

### Characterization of monoclonal antibody charge variant stability in CCCF

**Figure 1.**
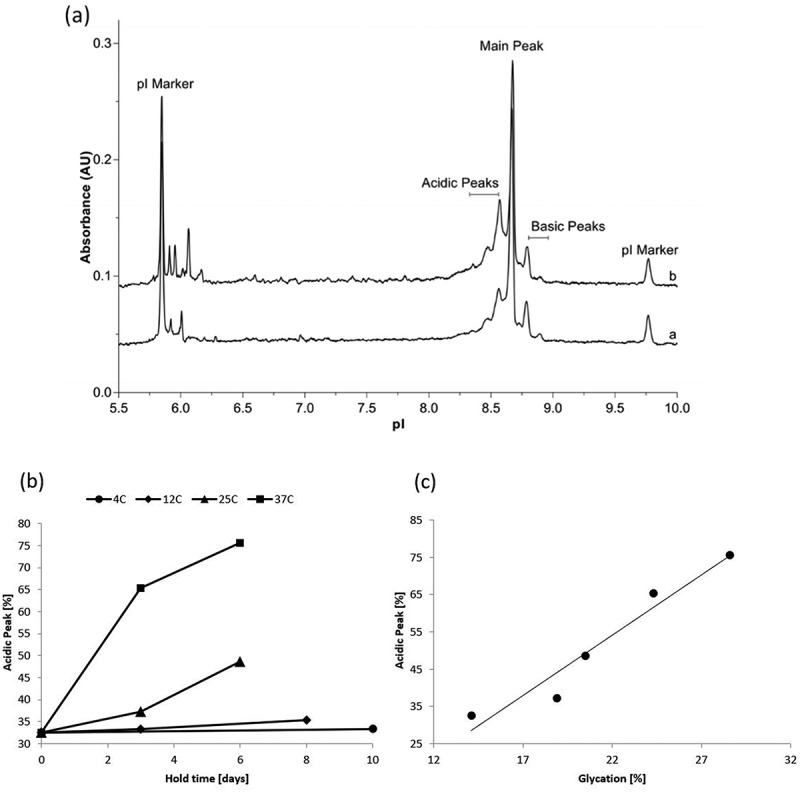
Changes in charge variant distribution during for mAb 1 during clarified cell culture fluid hold. a) Capillary Isoelectric Focusing results before and after 6 day hold at 25°C b) acidic peak (%) by Capillary Isoelectric Focusing as a function of CCCF hold time and temperature. (circle – 4°C, diamond – 12°C, triangle – 25°C, square −37°C) c) Correlation between acidic peak (%) by Capillary Isoelectric Focusing and glycation (%) by Quadrupole time-of-flight (Q-TOF) mass spectrometry.

CCCF containing mAb1 was subjected to stability studies described in the materials and methods section. After Protein A capture, samples were analyzed by capillary isoelectric focusing to measure charge heterogeneity, peptide mapping through mass spectrometry analysis to identify post-translational modifications, and time-of-flight mass spectrometry to determine the total level of protein glycation. The cIEF results for day 0 and for material held at 25°C for 6 days shows a decrease in main and basic peaks and a corresponding increase in the acidic peaks as a result of holding the clarified cell culture fluid, as shown in Figure 1(a). The study was conducted at 4°C, 12°C, 25°C, and 37°C and the observed rate of change of the acidic peak was highly dependent upon the temperature at which the material was held, as shown in Figure 1(b). When stored at 37°C the acidic peak approached 80% within 6 days. This data is not unexpected as the cell culture harvest day has been shown to impact the charge variant distribution through increasing the antibody’s mean residence time in the media (data not shown). Therapeutic antibodies are susceptible to several modifications that change the overall charge of the molecule impacting its isoelectric point. As a result of these modifications, an antibody can be viewed as a heterogeneous population of molecules with differing isoelectric points. Capillary isoelectric focusing separates these molecules based on their isoelectric point generating a profile of the isoelectric point distribution. Molecules with higher net negative charge will result in a lower pI. Modifications that result in a more negative molecule include N-terminus cyclization of Glu or Gln to pyroglutamate (loss of a positive charge), and deamidation of Asn or Gln (introduction of a negative charge and glycation (loss of a positive charge) [12]. Another source of charge heterogeneity involves the processing of C-terminus Lys residues. During manufacturing of therapeutic antibodies, C-terminal Lysine residues are typically removed but this process can be incomplete. The presence of a lysine residue bestows a positive charge on the molecule. Antibodies that still possess either one or two C-terminal Lysine residues will have a more positive charge resulting in a higher pI (more basic pI). [12]

Alt text: A three panel figure where panel ‘a’ includes a graphical representation of a typical Capillary Isoelectric Focusing profile where the absorbance (y-axis) is plotted vs pI (x-axis) and Acid, Main and Basic peaks are identified. The ‘b’ panel shows the temperature dependence of % acidic peak (y-axis) vs the total time CCCF is held (x-axis). The ‘c’ panel shows a correlation between the %acidic peak (y-axis) vs % glycated species (x-axis).

To determine which of these modifications occurred during the stability study, peptide mapping through mass spectrometry analysis was conducted which assesses deamidation and the presence of C-terminal Lysine and Pyroglutamate. Table 1 shows only minor changes in these attributes were observed between the day 0 and day 6 samples. As the small increase in deamidation, and even smaller increase in total N-terminal pyroglutamate, could not be the cause for the nearly 40% increase in acidic peak observed upon storing clarified cell culture fluid at 37°C for 6 days, material from several time points were analyzed to determine the glycation levels using time-of-flight mass spectrometry. An increase in glycation was observed during both the room temperature and 37°C stability studies. As seen in Figure 1(c), this increase in glycation correlates well (correlation coefficient 0.971) with the observed increase in the acidic peaks as measured by capillary isoelectric focusing. This data indicates that the primary mechanism for the increase in acidic peaks is the glycation of the protein and not N-terminus cyclization or deamidation.

**Table 1.** Peptide mapping results for mAb1 before and after a 6 day hold at either 25°C or 37°C.

Sample Description	Total Deamidation (%)	Total C-terminal Lysine (%)	Total N-terminal Pyroglutamate
Day 0	2.9	5.9	2
Day 6 at 25°C	3.8	5.7	2.3
Day 6 at 37°C	6.5	5.6	4

### Assessing the prevalence of glycation on several other monoclonal antibodies

**Figure 2.**
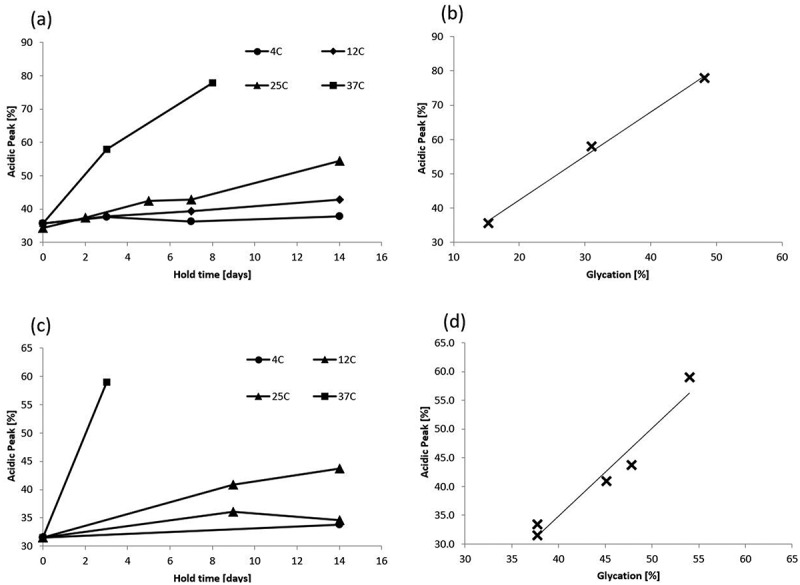
mAb2 and mAb3 changes in charge variant distribution during clarified cell culture fluid hold CIEF are similar to mAb1. Acidic peak (%) by Capillary Isoelectric Focusing as a function of CCCF hold time and temperature (circle – 4°C, diamond – 12°C, triangle – 25°C, square −37°C)for mAb 2 (a) and mAb 3 (c). Correlation between acidic peak (%)by Capillary Isoelectric Focusing and glycation (%) by Quadrupole time-of-flight (Q-TOF) mass spectrometry for mAb2 (b) and mAb 3 (d).

To determine whether the previously described observations are specific to mAb1 or whether they apply more generally to other therapeutic monoclonal antibodies, similar clarified cell culture fluid stability studies were conducted on two additional antibodies (mAb2 and mAb3). Figure 2 shows a similar pattern of charge variant changes as a function of temperature and hold time for both mAb2 and mAb3 as was seen for mAb1. The time-of-flight mass spectrometry analysis indicates that glycation level correlates well with the acidic peak percentage for both mAb2 (correlation coefficient 0.998) and mAb3 (correlation coefficient 0.974) as seen in Figure 2(b, d), respectively.

Alt text: A four panel figure where panels ‘a’ and ‘b’ show the temperature dependence of % acidic peak (y-axis) vs the total time CCCF is held (x-axis) and the correlation between the %acidic peak (y-axis) vs % glycated species (x-axis) for a second mAb. Panels ‘c’ and ‘d’ show the temperature dependence of % acidic peak (y-axis) vs the total time CCCF is held (x-axis) and the correlation between the %acidic peak (y-axis) vs % glycated species (x-axis) for a third mAb.

### Identification of reactive lysine residues on 3 model monoclonal antibodies

Having shown that charge variant distribution is sensitive to the time and temperature of the CCCF hold for three different monoclonal antibodies and that these changes correlate with the overall levels of protein glycation, we next aimed to determine if the sites of the sugar addition were random or specific and, if specific, were these sites similar across all three antibodies. For each antibody, the starting material and the last time point gathered during the 37°C arm of the stability study were subjected to peptide sequencing by mass spectrometry. By comparing the percentage of molecules glycated at each reactive residue at time zero and day 6 we are able to determine which sites are susceptible to glycation during the clarified cell culture fluid hold. This allows us to focus our efforts on those loci that are likely responsible for the change in charge variant. Table 2 summarizes those lysine resides who glycation level increased over the stability studies. The following lysine residues, present in the conserved region of these monoclonal antibodies, showed increased levels in 2 of the 3 antibodies tested: light chain K183, light chain K188, and heavy chain K326. Interestingly, several mAb3 variable region lysine residues were found to be susceptible to glycation. Both light chain lysine residues identified (K188 and K183) have previously been identified as glycation sites Quan et al. [9] utilized borate affinity purification to enrich for glycated molecules and identified eight glycated lysine residues with relative levels of glycation between 1% and 12%. Interestingly, Quan’s data indicated that K188 and K183 were lysines with some of the highest levels of glycation (12% and 6% respectively). Taken together, our results and the Quan study indicate that lysine glycation occurs at specific sites as opposed to randomly over all lysine residues.

**Table 2.** Relative increase in glycation level after clarified cell culture fluid hold at 37°C.

Sample Description	Relative Increase From Day 0 to End of 37°C Hold		
	Mab #1	Mab #2	Mab #3
LC3 (variable region)	NA	NA	107.1
LC5 (variable region)	NA	NA	29
LC6 (K***)	NA	NA	37
LC8 (K183)	105.8	175	46
LC9 (K188)	97.2	NA	81.3
HC4 (variable region)	NA	NA	77.5
HC5 (variable region)	NA	NA	71.9
HC6 (variable region)	NA	NA	95.9
HC9 (K***)	NA	160	NA
HC12 (K326)	166.1	183.3	77.8

### Protein mutagenesis to eliminate reactive lysine residues and improve charge variant stability on a model monoclonal antibody

**Figure 3.**
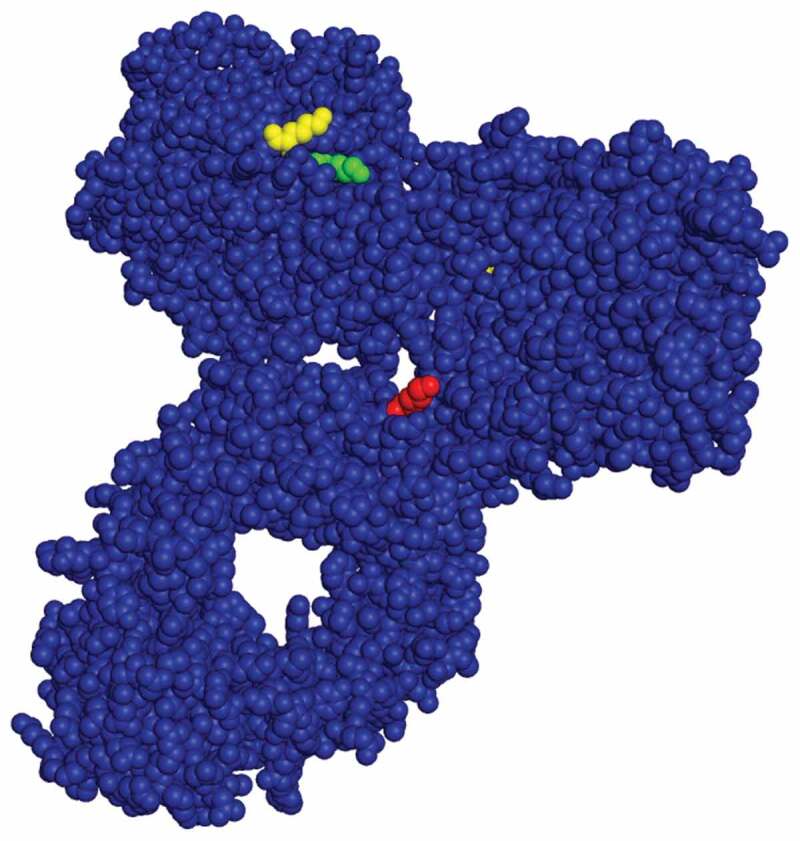
Crystal structure of an IgG with LC K188 (yellow), LC K183 (green) and HC K326 (red) highlighted (1HZH – https://www.rcsb.org/structure/1HZH).

We surmised that if glycation occurs at specific lysine residues, due to solvent accessibility, local amino acid sequences or some combination of both, then elimination of one or more of these reactive lysine residues should mitigate the change in charge variant distribution observed of the CCCF holds. mAb 3 was selected for further study. Three conserved lysine residues showed an increase in glycation of the CCCF hold for at least two of the three mAbs studied. These residues are LC8 (K183), LC9 (K188) and HC12 (K326). In Figure 3, a crystal structure of human IgG (1HZH) [15] is presented with these three residues highlighted in green (K183), yellow (K188) and red (K326). All three residues are on the surface of the antibody which indicates that solvent accessibility plays a key role in which lysine residues are glycated. In the mutant studies, these lysine residues were replaced with arginine because they are both positively charged amino acids of similar size. Table 3 shows the four mutants generated. Mutant 1 contains the K188R mutant. Mutant 2 contains a K326R mutant. Mutant 3 contains both the K183R and K188R mutations. Mutant 4 contains the K183R, K188R and K326R mutations.

Alt text: A image showing the crystal structure of a typical IgG with several reactive lysine residues highlighted showing proximity to the surface of the molecule.

**Table 3.** mAb3 mutants generated for this study.

Sample Designation	Mutations
mAb3	NA
Mutant 1	LC K188R
Mutant 2	HC K326R
Mutant 3	LC K183R and K188R
Mutant 4	LC K183R, K188R and HC K326R

The mutants were generated through an overlap extension PCR and cloned into a mammalian expression vector with unique restriction sites. Chinese hamster ovary cells were transiently transfected and cultured with enriched cells were harvested and supernatant was collected 14 days post transfection.

**Figure 4.**
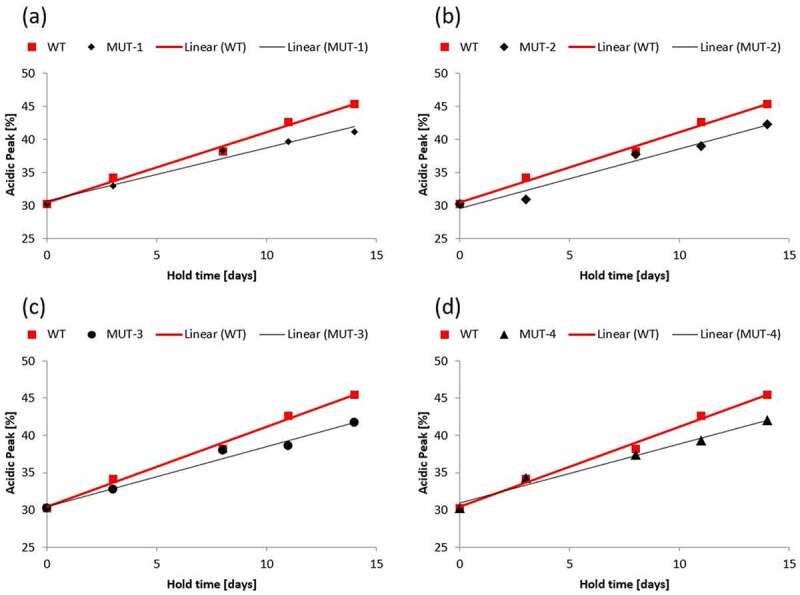
Acidic peak (%) by capillary isoelectric focusing as a function of CCCF hold time and temperature (circle – 4°C, diamond – 12°C, triangle – 25°C, square −37°C) for mAb 3 Mutants.

CCCF containing either mAb3 or one of the mutants was subjected to stability studies described in the materials and methods section. After Protein A capture, samples were analyzed by capillary isoelectric focusing to measure charge heterogeneity. Figure 4 shows the % acidic peak normalized to the mean starting acid peak value for mAb3 and all mutants studied. Normalization of the data was performed because small differences observed in the starting acidic peak percentage made visual comparisons of different mutants difficult. While mAb3 showed an increase in acidic peak percentage of 1.1% per day, all the mutants tested showed a significantly smaller rate of change. Mutant 1 had an increase in acidic peak of 0.8% per day which is a 27% reduction as compared to mAb3.

Alt text: A four panel figure where each panel shows the % Acid peak (y-axis) vs hold time (x-axis) for four different mutants.

### Assessing impact of K188R mutation on protein stability and function

**Figure 5.**
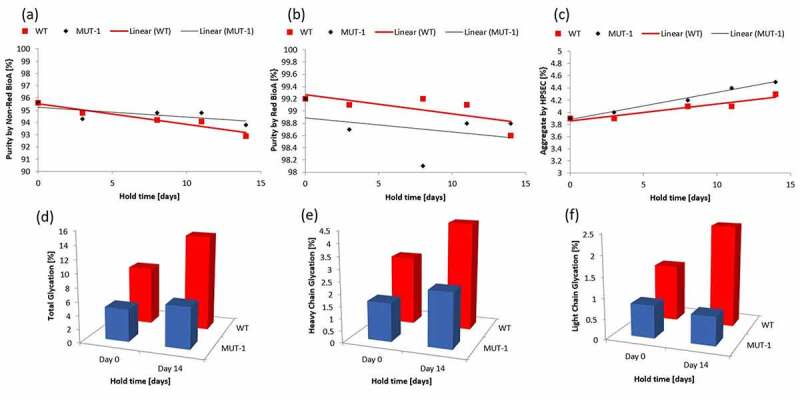
Characterization results for mutant 1. Total purity by Non-reduced (a) and Reduced (b) BioA show no significant difference in stability between mAb3 and Mutant 1. Rate of aggregate formation (c) is slightly higher for Mutant 1. Presented are amount of total glycation (d), heavy chain glycation (e) and light chain glycation (f) during clarified cell culture fluid holds for both mAb3 and Mutant #1. The total glycation and light chain glycation are reduced which is consistent with the targeted mutation of a single light chain lysine.

Having shown that mitigation of glycation-driven changes in charge distribution can be achieved through the mutation of reactive lysine residues the next step was to assess any additional impacts on function or stability as a result of these mutations. Mutant 1 was selected for further study. CCCF containing mAb3 or Mutant 1 was subjected to stability studies described in the materials and methods section. After Protein A capture, samples were analyzed by time-of-flight mass spectrometry (to assess glycation levels), non-reduced capillary electrophoresis (to assess fragment levels), high performance size-exclusion chromatography (to assess aggregate levels), and microneutralization activity assay (to assess antibody potency). Figure 5 summarizes the results obtained from this study. Glycation data indicates that the overall glycation level of the light chain was stable while the heavy chain showed an increase in glycation over the hold. This data is expected as mutant #1 only contained a single mutation on the light chain. No significant difference was observed in the rates of fragmentation between the mAb3 and Mutant #1. A small difference in aggregate formation was observed with Mutant #2 having a rate of formation of 0.04%/day vs 0.03%/day for the mAb3. This indicates that the *K188R* mutation in mutant 1 did not have a significant negative impact on other stability indicating attributes. Table 4 shows the results of the microneutralization assay which indicates that the mutation does not significantly impact potency and that even after 14 days at 25°C the mutant is as stable as the mAb3 molecule from a potency perspective.

Alt text: A 6 panel figure where panels ‘a’, ‘b’, and ‘c’ show impurity levels as measured by either non-reducing gel electrophoresis, reducing gel electrophoresis, or High-Performance Size-Exclusion Chromatography vs hold time (x-axis) for a single mutant. Panels ‘d’, ‘e’ and ‘f’ are bar graphs that the relative level of glycation in the wild type mAb vs the mutant where ‘d’ shows total glycation level, ‘e’ shows heavy chain glycation level, and ‘f’ shows Light chain glycation levels.

**Table 4.** Bioassay results for mAb3 and mutant 1, before and after 14 day hold at 25°C.

Sample Description	Sample Concentration (mg/ml)	Assayed % Relative Potency		
		Analysis #1	Analysis #2	Mean
mAb3 – Day 0	2.5	109.5	131.1	119.8
mAb3 – Day 14	2.5	111.9	125.2	118.4
Mutant 1 – Day 0	2.5	90.1	98.1	94
Mutant 1 – Day 14	2.5	92.7	104.9	98.6

## Conclusions

The work presented here describes the identification of conserved lysine residues present in several therapeutic monoclonal antibodies that are susceptible to glycation when held in clarified cell culture fluid. We have shown that elimination of either light chain K188 or heavy chain K329 from the conserved region of a monoclonal antibody can improve charge variant stability in clarified cell culture fluid up to 20%. We further demonstrated that replacing K188 with arginine does not have a negative impact upon other stability indicating attributes or antibody function.
